# Future Bloom and Blossom Frost Risk for *Malus domestica* Considering Climate Model and Impact Model Uncertainties

**DOI:** 10.1371/journal.pone.0075033

**Published:** 2013-10-08

**Authors:** Holger Hoffmann, Thomas Rath

**Affiliations:** Biosystems Engineering, Institute for Biological Production Systems, Leibniz Universität Hannover, Hannover, Germany; Plymouth University, United Kingdom

## Abstract

The future bloom and risk of blossom frosts for *Malus domestica* were projected using regional climate realizations and phenological ( = impact) models. As climate impact projections are susceptible to uncertainties of climate and impact models and model concatenation, the significant horizon of the climate impact signal was analyzed by applying 7 impact models, including two new developments, on 13 climate realizations of the IPCC emission scenario A1B. Advancement of phenophases and a decrease in blossom frost risk for Lower Saxony (Germany) for early and late ripeners was determined by six out of seven phenological models. Single model/single grid point time series of bloom showed significant trends by 2021–2050 compared to 1971–2000, whereas the joint signal of all climate and impact models did not stabilize until 2043. Regarding blossom frost risk, joint projection variability exceeded the projected signal. Thus, blossom frost risk cannot be stated to be lower by the end of the 21st century despite a negative trend. As a consequence it is however unlikely to increase. Uncertainty of temperature, blooming date and blossom frost risk projection reached a minimum at 2078–2087. The projected phenophases advanced by 5.5 d K^−1^, showing partial compensation of delayed fulfillment of the winter chill requirement and faster completion of the following forcing phase in spring. Finally, phenological model performance was improved by considering the length of day.

## Introduction

Apple production and its economic efficiency are clearly influenced by blossom frosts [1]. In addition, global warming could increase the risk due to greater changes in the date of flowering than in the last spring freeze or increasing variability in both. A generally higher risk of frost after bud burst for warmer winters was further stated as due to faster completion of the chilling requirement [2]. Past observations of late frosts and blossom frosts around the world have indicated a decreasing [3], [4] up to increasing risk [4]–[8] for fruit trees. However, findings cannot be generalized as they vary regionally. For instance, observed damages due to late frost increased in Northern Japan while other regions of Japan exhibited different tendencies [4]. An analysis of meteorological and phenological records of the Rhineland fruit-growing region in the West of Germany revealed, that risk of apple yield loss due to frosts in April remained unchanged during the period 1958 to 2007 [9]–[11]. This is consistent with studies showing an advance during the past of about 2.2 d/decade for both the last spring freeze (≤0°C, Central Europe, 1951–1997) [12] and for apple flowering (BBCH 60 [13], Germany, 1961–2000) [14].

Regardless of its development during the past, future blossom frost risk development remains uncertain as published estimates diverge (Table 1). Discrepancies are mainly due to differences in selected regions and varieties, as well as to the fact, that blossom frost risk computation requires estimates for flowering dates in addition to consistent climate time series which reproduce temperature thresholds (e.g. 0°C ) accurately. For this purpose climate model temperature time series are used as input for empirical phenological models accounting for chilling and/or forcing phases in winter and spring respectively [15]. While most climate scenarios describe an enhanced warming beyond 2040 [16], the following risk estimates are given. For the apple cultivar *Golden delicious* a “decreasing trend … of little significance” was found (Trentino, Italy), concluding that blossom frost risk “will not differ greatly from its present level” [17]. Similarly, for Finland the risk is expected to generally “stay at the current level or to decrease” for the period 2011–2040 compared to 1971–2000, excepting the southern inland which exhibits increases [18]. Increases in frost damage to apple blossom (*Malus pumila* Mill. cv. Cox’s Orange Pippin) were estimated for Britain [19] and an increase in the frequency of apple blossom frost damage was projected for Saxony (East Germany) by applying a simple thermal model to predict flowering, beginning on each 1 January [20]. Using the same approach, no increase in the mean apple blossom frost risk for Lower Saxony (Saxony and Lower Saxony are non-adjacent states) was found [21], despite temporarily/regionally increasing blossom frost risk.

**Table 1:** Published projections of future apple blossom frost risk.

Region	Increase (+)Decrease (−)No change (°)	Model	Statistics ontime series^a^	Ref.
Trentino, Italy	−, °^b^	Modified Utah	yes	[17]
Finland	−, °,+^b^	Thermal Time	no	[18]
Britain	+	Thermal Time-Chilling	no	[19]
Saxony, Germany	+	Thermal Time	no	[20]
Lower Saxony, Germany	−, °,+^b^	Thermal Time	yes	[21]

a Tests on blossom frost risk.

b depending on subregion.

These differences in estimates can be attributed to two deficits:

1. The modeling properties of the mentioned model [20], [21] are very limited for climate impact studies, as it solely calculates the onset of a phenophase based on accumulation of a heat requirement (forcing), hence assuming that dormancy has already been satisfied by a fixed starting date (see [22] for more details). Since future fulfillment of dormancy cannot be guaranteed, models including chilling phases seem to be more suitable for future climate impact simulations [23]. With their help, a possible impact of climate change on the fulfillment of dormancy [6] can be assessed. However, most of these models rely only on air temperature, ignoring possible influences of other climatic variables. Nevertheless improvement was found after including light conditions in the form of day length [24], [25], despite ongoing discussions about the influence of light conditions on tree phenological phases [26].
2. Published estimates of future blossom frost risk (Table 1) are based on single climate realizations and out of five studies, only two presented statistics for future blossom frost risk [17], [21]. However, assessing climate impact on the basis of models involves error concatenation resulting from the following chain of information. The future climatic impact is studied with the help of simulated climate time series, generated by global circulation models (GCM) and regionalized or downscaled by regional climate models (RCM). For this purpose these climate models are forced with greenhouse gas emissions scenarios of an evolving world (IPCC scenarios, SRES emission scenarios, [16], [27]). In order to estimate climate projection uncertainty, ensembles of GCM-RCM combinations or several realizations of one GCM-RCM combination (runs) are usually produced. These climate time series are used after down-scaling to drive impact models in order to assess the climatic impact in such different fields as coastal protection, water management, environmental research, food supply, urban planning and land use. Since models cannot reproduce every environmental aspect in real accuracy and resolution, systematic deviations of simulated and observed climate time series as well as of simulated and observed climate impact have to be taken into account. Depending on model sensitivity and question at hand, these biases can be removed by bias correction (e.g. 1-dimensional [28]; 2-dimensional [29]). Hence the chain of information for climate impact is: Scenario - emission - GCM - RCM - climate run - (bias correction) - impact model. Further chain members (e.g. prevention, adaptation strategies) or influences (e.g. feedbacks, interpolation, statistics) are possible. Since each member of this chain exists in different versions, numerous computations have to be conducted in order to cover the whole set of information available. Therefore most impact studies focus on “likely” scenarios [30], often not considering the full range of possibilities. This leads to the effect of possibly biased but significant trends of single or similar time series.

Taking these deficits into account, the objective of this work is to present a robust estimate of future blossom frost risk, taking the climate-model-impact-model uncertainty into account, including two new developed extensions of one sequential and one parallel chilling-forcing model considering light conditions.

## Methods

### General Procedure and Regional Focus

Thirteen simulated time series of air temperature from varying regional climate models were used to drive seven phenological models for the projection of apple bloom in Lower Saxony, Germany, whereas blossom frost risk was obtained by evaluating the temperature following bloom. Changes of these variables over time and compared to a reference period are referred to as “signal” in the following. The behavior of signal and variance across climate and impact models was analyzed subsequently, extracting the fractional uncertainty (inverse of signal-to-noise ratio). From this the meaningful horizon of projection was obtained, being basically the year at which the investigated signal exceeds the variation of the signal. This climatological approach [31], [32] originally divides time series into their internal variability, scenario and model uncertainty. Advancing this approach beyond climatology, the present work estimates the extension of uncertainty from the climate signal to the climatic impact by dividing time series into their internal variability, climate model and impact model uncertainty of one scenario.

In order to project apple bloom, phenological models were calibrated with measurements of daily air temperature and observations of phenophases. Subsequent projection of future apple bloom was carried out with bias-corrected climate projections from physical-dynamical regional climate models (Table 2). Calibrated models were validated for accuracy in prediction of bloom by cross-validation as well as testing for different locations. Blossom frost risk estimates were validated first by calculating the accuracy of the phenological model (comparing measured blossom frost risk with blossom frost risk simulated with measured temperature) and secondly through calculating the influence of the time series on blossom frost risk projection accuracy (comparing simulated blossom frost risk from measured temperature with that from simulated temperature).

**Table 2:** Overview of employed data.

Data	Specification	Climate model runs	Resolution (spatial, temporal)	Period	Ref.
observed	early ripeners, BBCH 60		0.116°, d	1991–2012	a
flowering	early ripeners, BBCH 65		0.116°, d	1991–2012	a
(DOY)	late ripeners, BBCH 60		0.116°, d	1991–2012	a
	late ripeners, BBCH 65		0.116°, d	1991–2012	a
measured *T* (°C )^b^	115 stations		0.126°, d	variable	c
simulated		1. EH5-REMO5.7, C20 1/A1B 1^d^	0.088°, h	1951–2100	[58]
*T* (°C )^b^		2. EH5-REMO5.8, C20 1/A1B 2^e^	0.088°, h	1961–2100	[59]
		3. EH5-REMO2008, C20 3/A1B 3^f^	0.088°, h	1950–2100	f
		4. EH5-CLM2.4.11 D2 C20 1/A1B 1	0.165°, 3 h	1961–2100	[60]
		5. EH5-CLM2.4.11 D2 C20 2/A1B 2	0.165°, 3 h	1961–2100	[61]
		6. C4IRCA3_A1B_HadCM3Q16	0.223°, d	1951–2099	[62]
		7. CNRM-RM5.1_SCN_ARPEGE	0.232°, d	1951–2100	[62]
		8. DMI-HIRHAM5_BCM_A1B	0.223°, d	1961–2099	[62]
		9. DMI-HIRHAM5_A1B_ARPEGE	0.223°, d	1951–2100	[62]
		10. DMI-HIRHAM5_A1B_ECHAM5	0.223°, d	1951–2099	[62]
		11. ICTP-REGCM3_A1B_ECHAM5_r3	0.232°, d	1951–2100	[62]
		12. KNMI-RACMO2_A1B_ECHAM5_r3	0.223°, d	1951–2100	[62]
		13. MPI-M-REMO_SCN_ECHAM5	0.223°, d	1951–2100	[62]

a German Meteorological Service. Phenological observation program. URL: http://www.dwd.de (April 20, 2013).

b air temperature at 2 m elevation.

c German Meteorological Service. Station network. URL: http://www.dwd.de (April 20, 2013).

d “UBA”-Run, experiments 6215/6221.

e “BFG”-Run, experiments 29001/29002.

f experiments 1518/1518, Max Planck Institute for Meteorology, Hamburg, Germany.

### Climatic Data and Models

#### Data sources

**Figure 1:**
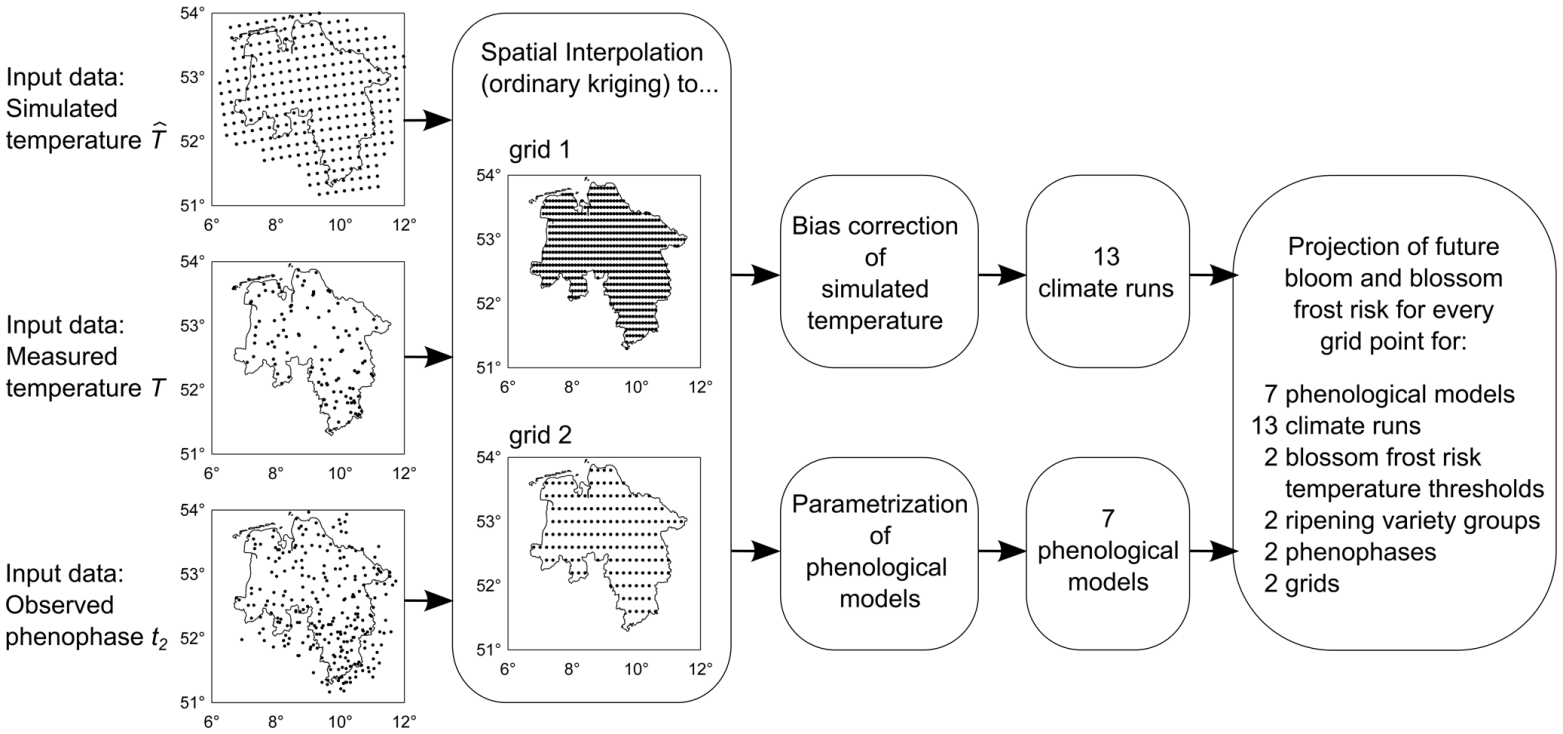
Scheme of used input data and projection. Note that for simulated temperature the grid of the regional climate model CLM is shown exemplarily.

Measured as well as simulated air temperature time series for Lower Saxony, Germany, (Table 2, Figure 1) were processed and applied as follows. Simulated temperature of regional climate model projections of the IPCC-emission A1B [27] was obtained from the Max Planck Institute for Meteorology, Hamburg, Germany, (in the following climate runs 1–5) and from the ENSEMBLES project (in the following climate runs 6–13).

**Temporal interpolation.** Temporal interpolation of measured daily temperature time series was used to obtain hourly time series, following a stepwise procedure of spline interpolation [21]. Resulting hourly temperature time series showed a year-round mean error of −0.031 K h^−1^ and mean absolute error (MAE) of 0.448 C h^−1^ as well as an error of 0.587 hours of frost (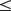
0°C) per month of April, compared to measured hourly time series at 56 sites. Time series of the climate model CLM (3 h resolution) were brought to hourly resolution by applying cubic spline interpolation.

#### Spatial interpolation

Spatial interpolation through ordinary kriging [33] was used to bring measured as well as simulated data to common and regular grids (0.1°⋅0.1° as well as 0.2°⋅0.2°) for the area 51° to 54° latitude north and 6.5° to 12° longitude east. While measured data was interpolated directly, simulated hourly temperatures (climate runs 1–5) were previously aggregated by taking the mean of each hour of nine neighboring model grid points (area approximately 30 km⋅30 km for REMO). By doing so for every model grid point and hence obtaining a spatial floating mean, the original model resolution was maintained. Simulated daily mean and minimum temperature time series were not aggregated due to the coarser spatial resolution.

#### Bias correction

Since several climate models underestimate the occurrence of frosts, simulated temperature series were bias-corrected for each month by distribution-based quantile mapping [28], using non-parametric transfer functions obtained by applying a Gaussian kernel with bandwidth h = 0.1 [34]. The period of comparison from which transfer functions were derived for bias correction was 54.4±7.3 years for climate runs 1 and 3, 49.8±4.9 years for climate runs 2, 4 and 5 as well as 57.9±4.4 years for climate runs 6–13 (mean ± standard deviation). Hence, the influence of the multidecadal variability was assumed to be negligible. Information on bias correction dynamics with climate runs 6–13 (Table 2) have been published [35].

#### Projection of temperature

In the following, temperature time series are presented as anomaly from the 1971–2000 mean as indicated by 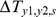 with the centers of the respective periods 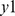 and 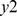 and grid points 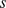 (see Methods S1 for equation).

#### Projection of last spring freeze

The last spring freeze was defined as the last day before July 31st, exhibiting a minimum air temperature ≤0°C, and taken directly for every year from temperature time series.

### Phenological Data and Models

#### Data sources

In order to simulate apple bloom phenophases, time series (Table 2, Figure 1) from the German National Meteorological Service (htp://www.dwd.de) of observed beginning of flowering (first flowers open) as well as onset of full bloom (50% of flowers open), defined as phenophases 60 and 65 on the BBCH-scale [13], were processed and used to calibrate phenological models for early and late ripening varieties as follows.

#### Spatial interpolation

Phenological time series were spatially interpolated as described above for measured temperature time series.

#### Basic phenological models

In principle, all applied phenological models (Table 3, 4, Methods S1) assume that the time of bloom is related to so-called sums of chilling and heat units (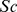, 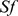) accumulated during winter (chilling phase) and spring (forcing phase), (see Table 4 for denominations). It is assumed, that 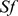 is related to 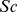 [36], [37]. The basic models (Table 3, models 1–4) have been described in the literature [17]–[21] and their equations are given in Methods S1.

**Table 3:** Phenological models.

No.	Type	Daylength	*Tbf*	*Tbc*	*Sf*(*t*_2_)	*Sc*(*t*_1_)	*t*_1_	*a*	b	c	Ref.
1	Thermal time	−	+	−	+	−	+^a^	−	−	−	[20]
2	Sequential chilling-forcing	−	+	+	+	+	−	+	+	−	[23]
3	Parallel chilling forcing	−	+	+	+	+	−	+	+	−	[23]
4	Modified Utah	−	+	+	+	+	−	−	−	−	[17], [43]
5	Thermal time	+	+	−	+	−	+	−	−	+	[25]
6	Sequential chilling-forcing	+	+	+	+	+	−	+	+	+	–
7	Parallel chilling forcing	+	+	+	+	+	−	+	+	+	–

a For model 1, t1 was set to January 1.

**Table 4:** Denomination of variables and parameters.

Notation	Description	Unit
*T*	Air temperature	°C
*Tbc*, *Tbf*	Base temperature for chilling, forcing	°C
*t*	Time	hour [h], day [d] or year [a]
*t*_0_	Start of the chilling period (dormancy)	day of the year [DOY]
*t*_1_	Chilling requirement completed, start of forcing	day of the year [DOY]
*t*_2_	Forcing completed (BBCH 60, BBCH 65)	day of the year [DOY]
*Sc*, *Sf*	State of chilling, state of forcing	–
*Rc*, *Rf*	Rate of chilling, rate of forcing	–
*D*	Daylength	h
*a*, *b*, *c*	Calibration parameters	–
*i*, *s*, *z*	Index variables	–
*θ*	Blossom frost risk	–
*β*	Temperature threshold for blossom frost	°C
*λ*	Parameter for calculation of mean and confidence level	–

#### Extended phenological models

Models including an additional day-length-parameter for the calculation of the forcing phase were included in the ensemble (Table 3, models 5–7), as a higher performance of model no. 5 has been reported. Models 6–7 are new model variations of the sequential and parallel chilling-forcing models [23], which were extended for a factor for the length of day 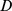, assuming that bloom is influenced by radiation only during the forcing phase. For both, the rate of forcing 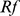 was calculated as follows:
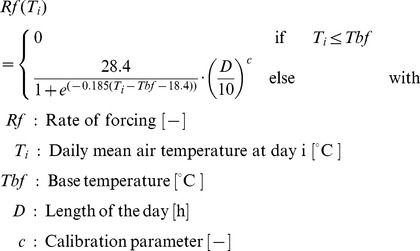
(1)


#### Parameter estimation and model validation

Models were parametrized for each grid point by fitting the models to observed bloom (BBCH-scale [13], stages 60 and 65 for early and late ripening varieties of *Malus domestica*) and measured daily air temperature (Table 2). Fitting was performed through bound-constrained simulated annealing, minimizing the root mean square error (RMSE) between observed and simulated day of the year (DOY) of bloom. Simulated annealing for parameter estimation of phenological models has been described in detail [38] and was performed in the present study by using the Global Optimization Toolbox (The Mathworks Inc., Natick, Massachusetts) on a computing cluster system (http://www.rrzn.uni-hannover.de/clustersystem.html). For this, 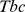 and 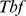 were searched between 0°C and 10°C, as this is believed to be the effective range of temperature on the development of apple trees [23]. The models were validated internally (same location) as well as externally (different location) by calculating the prediction root mean square error (PRMSE) determined by full-cross validation (“leave-one-out”) and by applying the model with optimized parameters to six different and randomly chosen locations in the range of 20 to 28.3 km distance.

All models accounting for 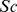 were initiated with 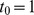 August. The simple thermal-time model (1) was started with fixed 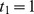 (January 1st, model 1), whereas the extended thermal-time model (5) was started on August 1st (DOY 213, 214) in order to optimize 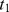. Models 1 and 5 do not account for a chilling phase and hence implicitly assume that chilling is already completed at 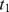.

### Projection of Bloom

Bias-corrected air temperature time series of 13 climate realizations (Table 2, Figure 1) were used as input for seven phenological models for 792 locations in Lower Saxony on a 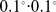 grid (climate runs 1–5) and for 274 locations on a 0.2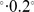 grid (climate runs 6–13, Table 2, Figure 1) to project future apple bloom. Projections were conducted for all grid points whereas presented results were restricted to the area of Lower Saxony (Figure 1) in order to avoid boundary effects due to interpolation. Comparison of results from all 13 projections took place on the grid of lower resolution. All simulations were conducted with early as well as late ripening varieties and for two phenological stages (BBCH 60, 65). The change in blooming date 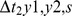 with the centers of the respective periods 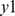 and 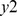 and grid points 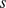 was calculated as the difference in the 30-year-mean for each grid point. Years with unfulfilled chilling were recorded by counting years without bloom or bloom projected for DOY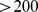 as fraction of occurrences in a 30-year-mean. Please see Methods S1 for equations.

### Projection of Blossom Frost Risk

Subsequently, years with occurrences of frosts (daily minimum temperature ≤0°C) and possibly blossom damaging situations (daily minimum temperature ≤2°C) during the time from simulated bloom (BBCH 60, BBCH 65) to the 31st of July of each year were counted separately. The additional threshold of 2°C was chosen in order to account for spatial discrepancies of observed bloom and measured temperature as well as for possible radiation frosts with tissue temperatures falling below air temperature [19], measured at standard meteorological conditions. Blossom frost risk was defined as the ratio of number of years with temperatures lower or equal to a predefined threshold occurring after a specific phenophase in 30 years:
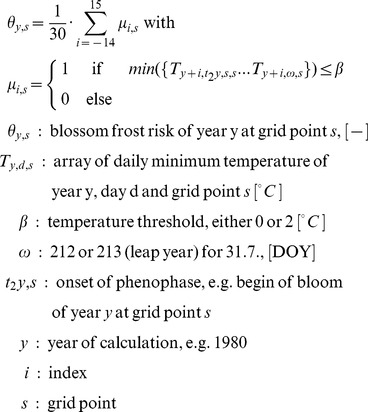
(2)


The change in blossom frost risk 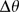 was calculated from 30-year-means of each grid point:
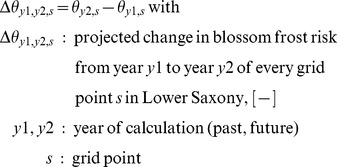
(3)


Probability mass functions were calculated in order to estimate the distribution of changes in blossom frost risk till the end of the 21st century (2070–2099 minus 1971–2000). The values of these probability mass functions were estimated non-parametrically with the help of kernel density estimation, applying a Gaussian kernel. Please see Methods S1 for equations.

### Partitioning of Uncertainty of Temperature, Bloom and Blossom Frost Risk

In order to estimate the meaningful projection horizon ( = ‘Time of emergence’, [39]) of the results obtained as described above, the fractional variance of the system was calculated and the total variance of the projection was partitioned. For this purpose the methodology of Hawkins and Sutton [31] was applied to the presented projections for the day of bloom 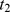. Instead of looking at different climate models and scenarios, the present work analyzes the internal variability, the uncertainty from climate realizations of one IPCC-scenario (A1B) and the variance resulting from the impact models. Impact models were weighted by their error as described for climate models [31]. The following calculations were carried out with 10 year mean moving average time series of the area mean of Lower Saxony (mean of all grid points 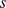, please see Methods S1 for equations). In brief, the total variance for bloom was calculated as described below. Projection uncertainty of temperature and blossom frost risk was calculated as described for bloom (temperature analysis only for internal and climate realization variability).
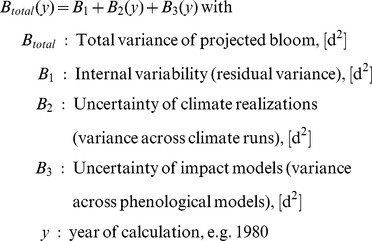
(4)


The contribution of 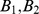 and 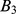 to the total variance can be expressed as fraction of the total variance:
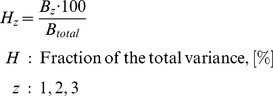
(5)


The mean change in blooming dates of all projections (climate impact signal) over the reference period was obtained as:
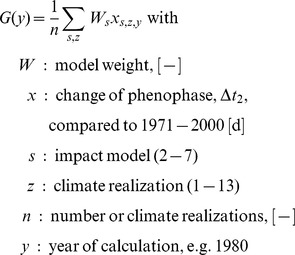
(6)


Models were weighted (eq. 6) with weights 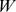 inversely proportional to their model error (see [31]), giving models with lower errors comparatively more importance. From 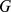 and 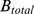 the fractional uncertainty 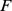, which is the inverse of the signal-to-noise ratio, was calculated as follows:
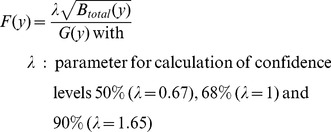
(7)


### Statistics of Single Time Series

Continuous time series of calculated completion of dormancy, blooming date and last spring freeze were analyzed using a Mann-Kendall-test [40], whereas trends in blossom frost risk were analyzed with a test by Cox & Lewis [41].

## Results

### Validation of Methods

The presented methodology was evaluated at the levels climate, quality of phenological model in order to simulate phenophases as well as blossom frost risk. A bias correction had no influence on the mean temperature pattern, whereas the accuracy of simulated frost distribution was drastically improved (Table 5), see also [35]). While climate model time series underestimated frosts in April, this was corrected through the bias correction.

**Table 5:** Stepwise error of simulation chain segments. SE: Simulation error, ABS: absolute level from measured data.

Parameter		*T* bias corrected	Frost occurrences permonth of April		Bloom^a^	Blossom^b^ frostrisk *θ*
			[h]	[d]	[d, DOY]	[−]
Frost	ABS	–	25	4	–	–
Frost	SE^c^	no	7	3	–	–
Frost	SE^c^	yes	<1	<1	–	–
Bloom	ABS	–	–	–	117–126	0.163
Bloom	SE^c^	no	–	–	–	–
Bloom	SE^c^	yes	–	–	4–8	–
Blossom frost	ABS	–	–	–	–	0.163
Blossom frost	SE^c^	no	–	–	–	–
Blossom frost	SE from phenol. models^cd^	yes	–	–	–	0.001–0.034
Blossom frost	SE from time series^ce^	yes	–	–	–	0.021–0.075

a min-to-max range across all ripening groups and phenophases.

b min-to-max range across all ripening groups, phenophases and phenological models.

c Mean absolute error (MAE), average over all grid points.

d Error from comparison of measured blossom frost risk with blossom frost risk simulated with measured temperature (1991–2012).

e Error from comparison of blossom frost risk simulated with measured temperature with blossom frost risk simulated.

with simulated temperature (1951–2012).

Models could be fitted to reproduce bloom with 3.2 to 5.7 d mean accuracy (RMSE), whereas testing models with fitted parameters (see Methods S1) for different locations revealed an external PRMSE of 3.9 to 8.0 d (Table 6). While the thermal time model (1) exhibited the highest mean error (1.8 d higher than mean of other models), the thermal time model with extension for day length exhibited the lowest mean error (2.0 d lower than mean of other models). On average models (1–3) were improved by 2.0 d when accounting for day length (models 5–7), whereas performance did not differ greatly between BBCH-stages 60 and 65 nor between early and late ripening varieties.

**Table 6:** Prediction Root Mean Squared Error PRMSE of phenological models [d].

Model	early ripeners		late ripeners		mean
	BBCH 60	BBCH 65	BBCH 60	BBCH 65	
1	7.97	7.26	7.28	7.27	7.45
2	6.67	5.95	6.24	6.03	6.22
3	7.10	6.30	6.54	6.25	6.55
4	6.81	6.83	6.54	6.67	6.71
5	4.14	4.12	3.91	4.34	4.13
6	4.96	5.08	4.88	5.10	5.00
7	5.13	5.19	4.89	5.29	5.13
mean	6.11	5.82	5.75	5.85	5.88

**Figure 2:**
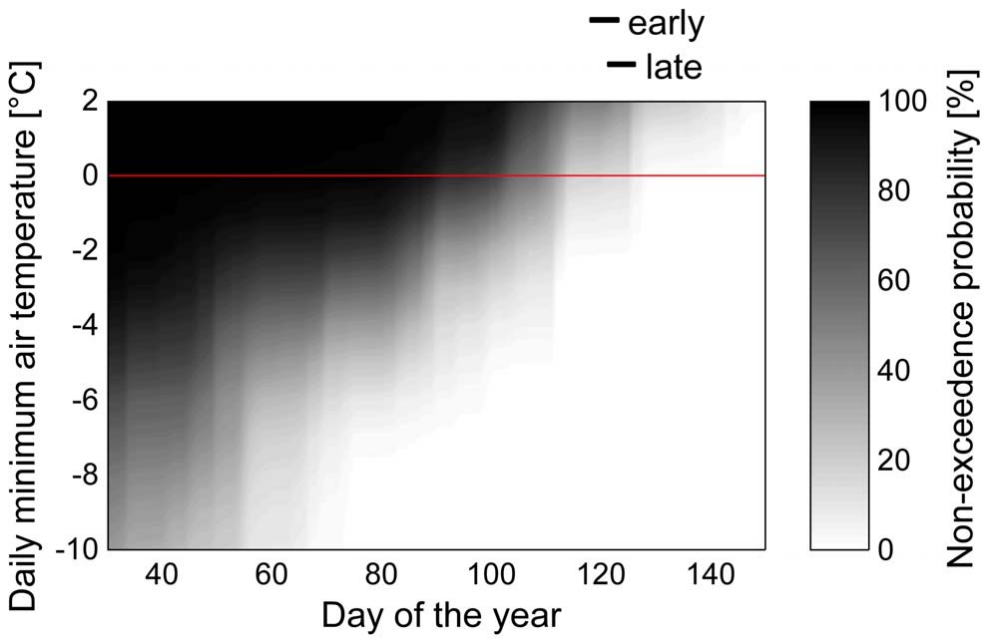
Present temperature incidence of Lower Saxony (1991–2010). Bars indicate mean flowering period (BBCH 60–65) of early and late ripening varieties.

Blossom frost projection accuracy was verified at different levels, since direct comparison of measured blossom frost with blossom frost from simulated time series is not possible in a direct manner for short periods (<30 a). Therefore the influences of phenological models and of time series on blossom frost incidents were extracted separately. Applying the phenological models to measured climate data of the calibration period 1991–2012 reproduced blossom frost incidences from measured temperature and measured bloom (Figure 2, Table 5). Subsequently the influence of the time series on blossom frost projection accuracy was tested by applying the validated phenological models on measured and on simulated-bias corrected time series (1951–2012). Despite bias correction, projection with simulated-bias corrected time series showed a mean absolute error (MAE) of blossom frost risk of up to 7.5 percentage points (Table 5). However, mean influences of impact model and time series on blossom frost risk projection accuracy were 1.4 and 3.6 percentage points respectively (mean MAE). Finally blossom frost risk was biased by +0.9 and −3.6 percentage points by impact model and time series, respectively, still resulting in an overall underestimation of blossom frost.

### Dormancy and Bloom

**Figure 3:**
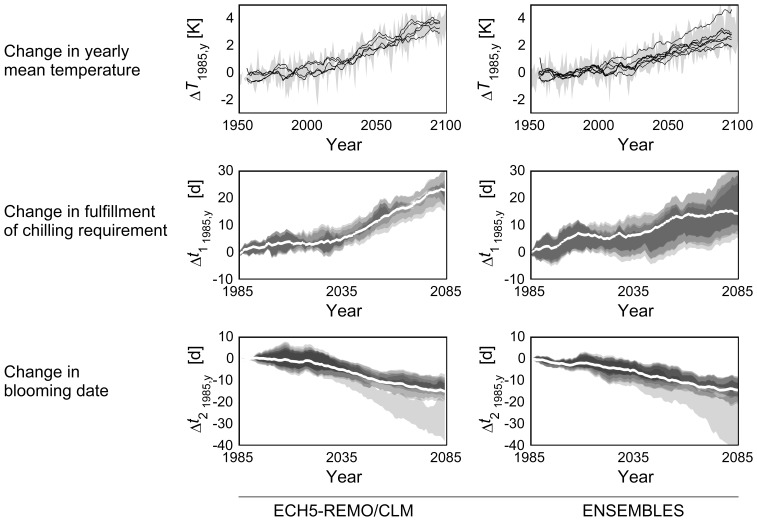
Projected changes in air temperature, fulfillment of chilling requirement and onset of flowering. Projected with 5 (ECH5-REMO/CLM) and 8 (ENSEMBLES) climate runs and five (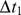) and seven (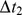) phenological models for Lower Saxony (area mean), relative to the 1971–2000 mean. 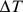: single year-mean, min-to-max range of climate runs (shaded area), 10 year moving average of each run (solid lines, see Methods S1 for equation). 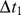, 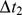: BBCH 65, early ripeners, 30-year-moving-average, all impact model mean (solid white line), single model range (shaded areas). The range of each phenological model (min-to-max) obtained from climate runs is plotted with 20% transparency (darker areas illustrate coinciding results).

**Figure 4:**
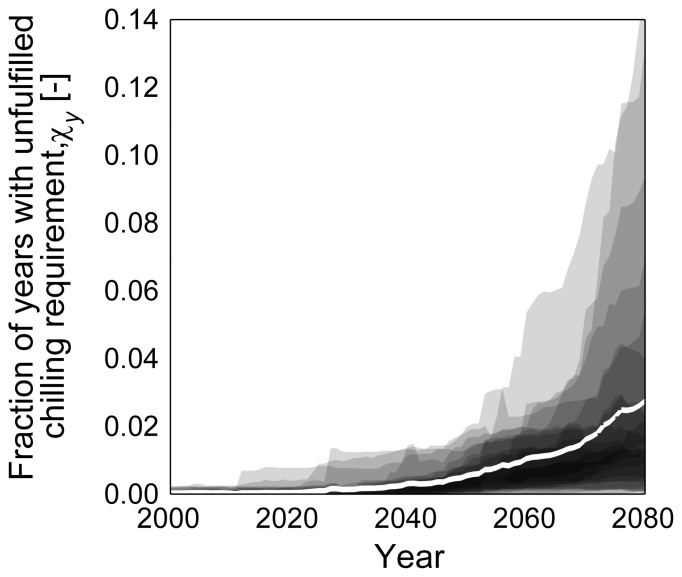
Proportion of years with unfulfilled chilling requirement. Areas: min-to-max range across seven phenological models for each climate run (area mean of Lower Saxony, 30-year moving average); white line: Mean of impact models and climate runs.

**Figure 5:**
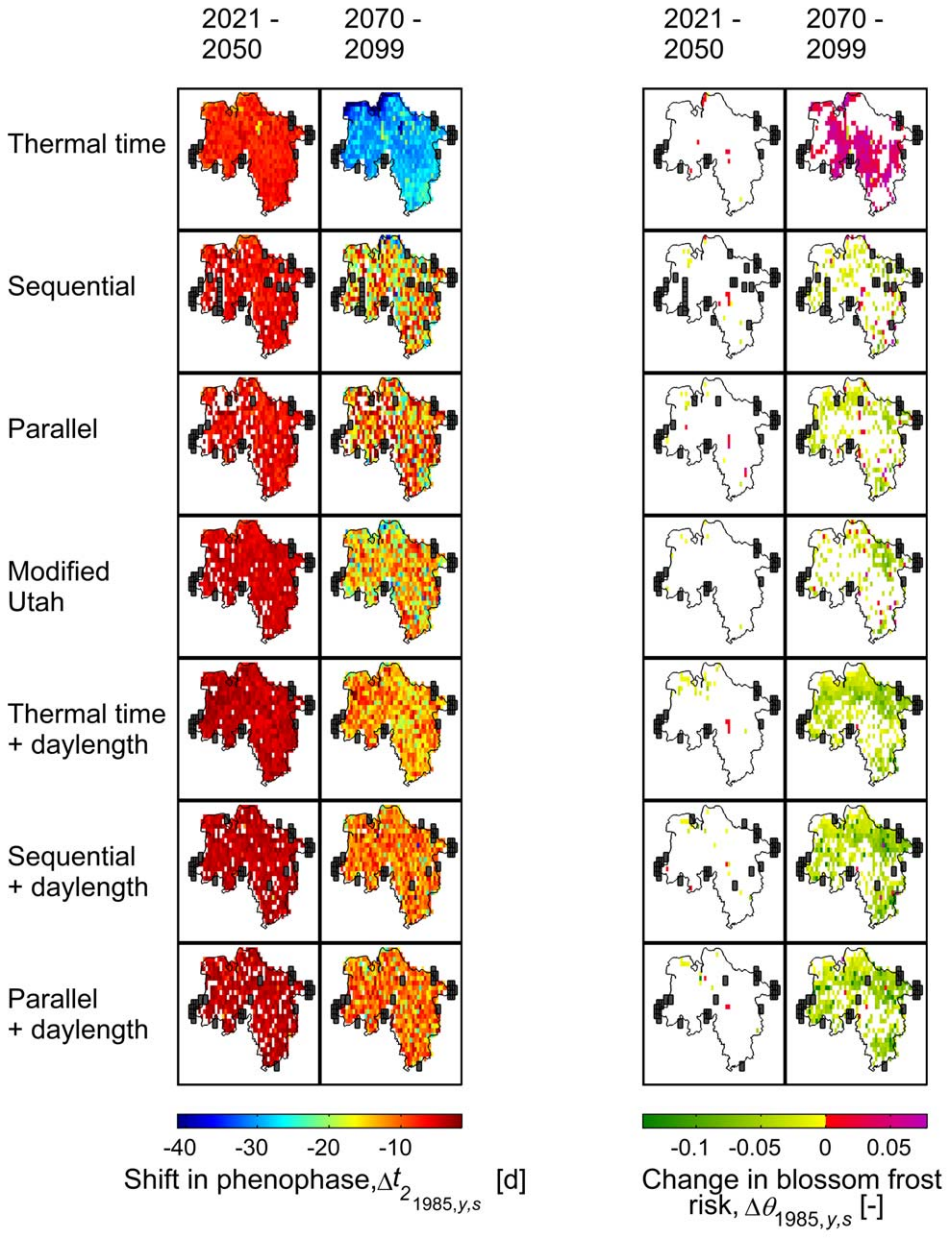
Changes in bloom and blossom frost risk as projected by different phenological models and climate runs 1–5. Early ripeners, BBCH 65, temperature threshold 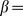
0°C, reference period 1971–2000, resolution 0.1°. White fields denote non-significant results, black fields denote missing/insufficient data. 1–99% percentile range. 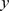 = 1985 and 2084, 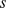 = grid point.

**Figure 6:**
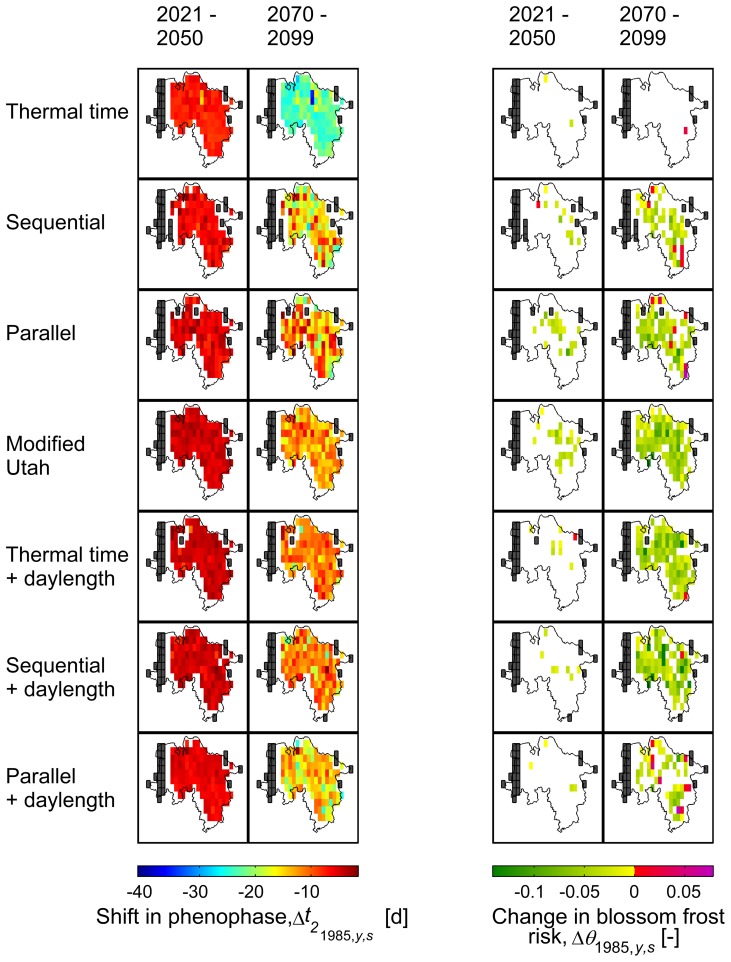
Changes in bloom and blossom frost risk as projected by different phenological models and climate runs 6–13. Early ripeners, BBCH 65, temperature threshold 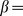
0°C, reference period 1971–2000, resolution 0.2°. White fields denote non-significant results, black fields denote missing/insufficient data. 1–99% percentile range. 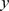 = 1985 and 2084, 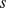 = grid point.

In the mean, observed bloom from 1991 to 2012 changed by −3.3 d K^−1^ (R^2^ = 0.87) while air temperature increased by 0.037 K a^−1^. Phenological models, which were calibrated with these data, gave the following results when applied to simulated temperatures. All chilling-forcing models consistently showed a delay for the release of dormancy 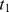 (Figure 3) with major changes not occurring before 2030, following the temperature warming patterns of both simulated climate data sets. However, 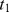 showed a larger spread across ENSEMBLES runs than for ECH5-REMO/CLM simulations, while the number of years with unfulfilled chilling requirement increased in both cases (Figure 4). Unlike 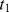, projection of the onset of the phenological phases for 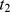 (BBCH 60, 65) revealed an advancement. While models 2–7 follow a relatively homogeneous pattern, model 1 projects a faster advance. These main patterns also become visible on a regional scale (Figure 5,6). However, changes in the day of bloom vary regionally depending on the model. Regarding the timescale, all models project a shift in the day of bloom of −5.4±3.0 d by 2035 compared to 1971–2000 (area mean, all varieties and stages), whereas results for 2084 differ. While model 1 shows the strongest change (−26.7±8.2 d), models 2–7 project a mean shift of approx. −12.9±3.3 days. The latter again differ in their regional variation. Although the classic sequential and parallel chilling forcing models (2–3) show a similar mean shift of bloom as their versions extended for daylength (models 5–7; −13.5 d and −11.2 d respectively), the former exhibit higher variation (±3.6 d and ±2.2 d respectively). A similar variation was also found for model 4 (±3.3 d).

### Projected Last Spring Freeze and Blossom Frost Risk

**Figure 7:**
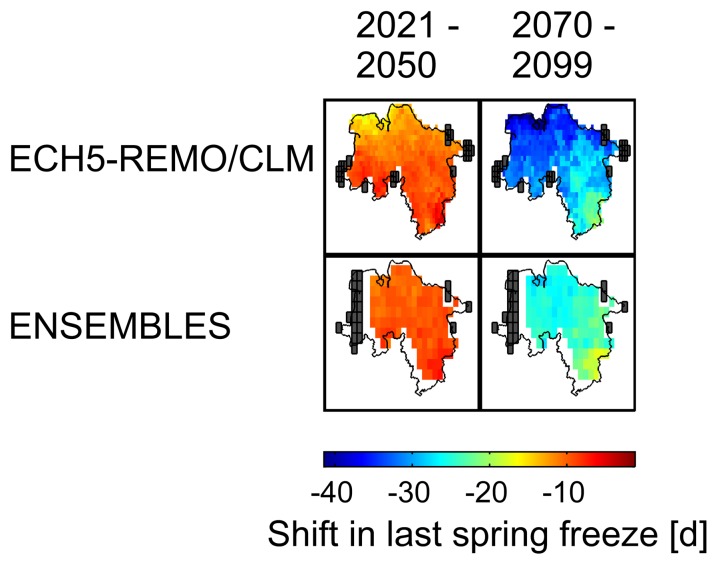
Changes in last spring freeze. Reference period: 1971–2000. White fields denote non-significant results, black fields denote missing/insufficient data. 1–99% percentile range.

**Figure 8:**
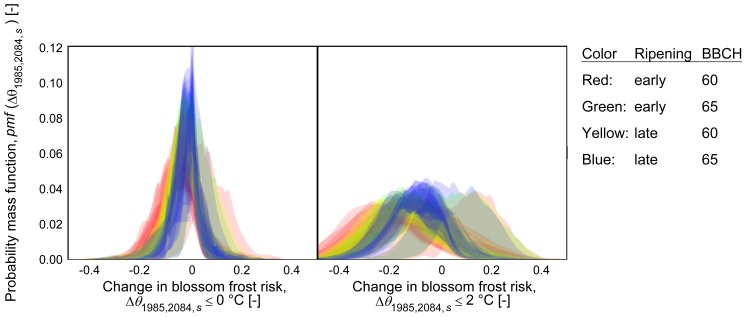
Distribution of projected changes in blossom frost risk by the end of the 21st century (2070–2099 minus 1971–2000) for early and late ripening varieties, phenophases BBCH 60 and 65 and 7 phenological models: Temperature thresholds 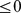
°C and 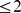
°C; inter-quartile range across 13 climate runs; phenological models are presented by same colors. Calculated from all grid points 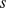 (see Methods S1 for equation).

According to the scenario and climate runs considered, the last spring freeze (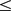
0°C) will shift by −10.0±4.2 days and −27.3±7.4 days by 2035 and 2084 respectively, with regard to the reference period 1971–2000 (Figure 7). Hence these 30-year-mean trends indicate an increasing discrepancy of the day of bloom and the last spring freeze. Correspondingly the mean occurrences of blossom frost (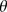) are projected to decrease in the long run (Figure 5,6). Nevertheless model 1, which showed the fastest advancement of bloom, projects a mean increase of blossom frost risk by 3.4 percentage points whereas models 2–7 project a mean change by −4.1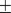 3.3 percentage points, ranging from −2.6 percentage points for late ripeners (BBCH 65) to −6.0 percentage points for early ripeners (BBCH 60). In the mean, runs of EH5-REMO/CLM and ENSEMBLES runs produced similar estimates for changes in blossom frost risk (−2.7±4.4 percentage points and −3.2±4.5 percentage points respectively). However, all models also exhibited regional and temporary increases in blossom frost occurrences. The resulting probability mass function values (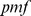) are shown in Figure 8, displaying also the contrary result of model 1. A larger spread and stronger decrease was observed for the probability of temperatures of 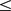
2°C after onset of phenophases.

### Projection Uncertainty

**Figure 9:**
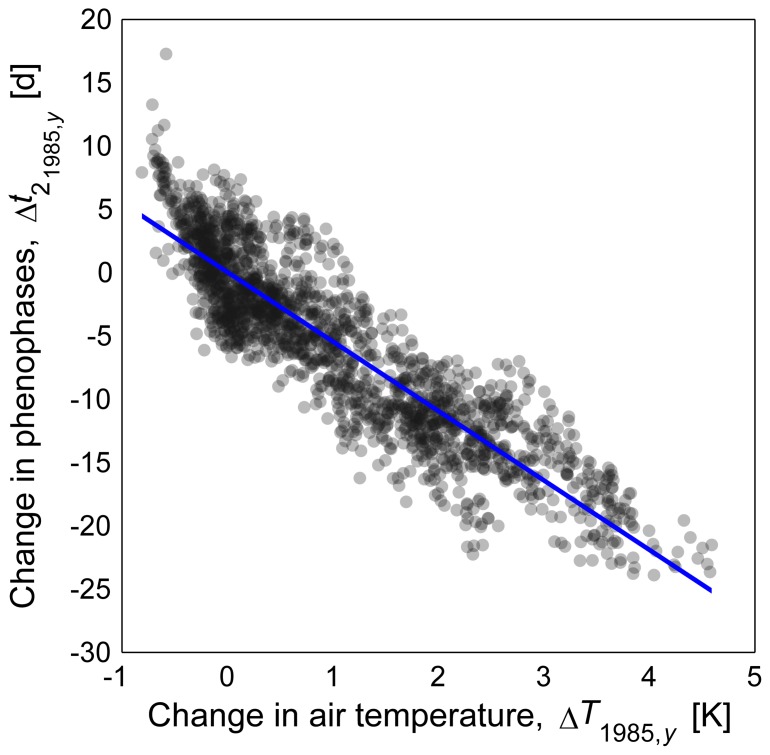
Simulated relation between projected absolute changes in decadal mean air temperature and changes in the day of bloom compared to the 1971–2000 mean. Depicted values are related to 139 years (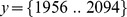, see Methods S1 for equation) and 13 climate realizations for the area mean of 2 phenophases and 2 variety groups. Slope of regression (solid line) = −5.4842, offset = 0.0385, R^2^ = 0.81.

Phenophases followed temperature patterns closely, with early and late ripening varieties advancing at 5.6 and 5.4 d K^−1^ respectively and BBCH 60 and BBCH 65 advancing at 5.6 and 5.4 d K^−1^ respectively, resulting in a mean change of −5.5 d K^−1^ (Figure 9). Higher correlations were found between changes in begin of flowering date and mean temperatures between February and April (−6.1 d K^−1^, R^2^ = 0.93). However no correlation was found between changes in the respective variances of temperature and flowering dates, with exception of the simple thermal time model (model 1, data not shown).

**Figure 10:**
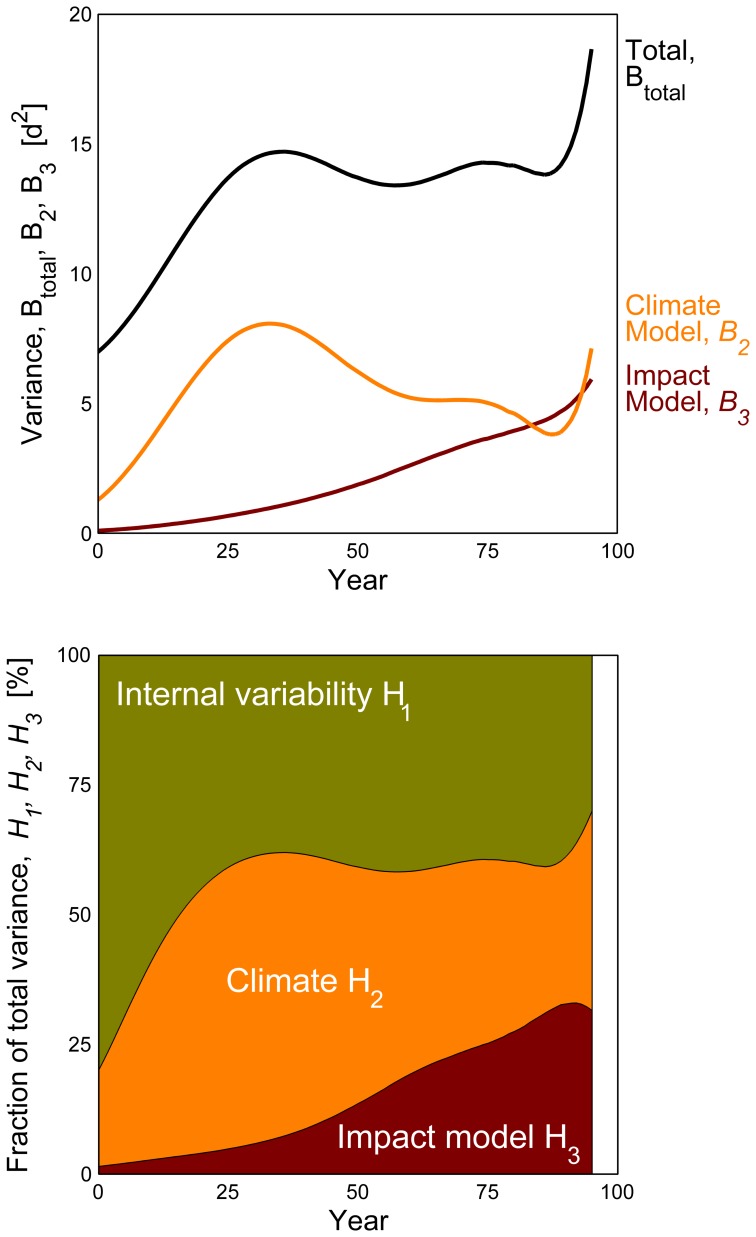
Uncertainty in the projection of apple bloom (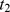). Drawn from phenological impact models 2–7 and 13 climate projections. Mean uncertainty of phenophases (BBCH 60, 65) and ripening groups (early, late).

The projection uncertainty increased with increasing lead time (Figure 10, top) and for the period investigated, the accuracy of the projection of 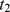 in the short run is mainly dependent on the projected climate and internal variability. With increasing horizon of projection, the climate signal (temperature) becomes stable while impact/phenological model results diverge. Consistently fractions of climate and internal variability of the total variance decreased with increasing lead time (Figure 10, bottom). Finally, the projection accuracy at the end of projection horizon depended equally on the climate and impact/phenological model variance.

**Figure 11:**
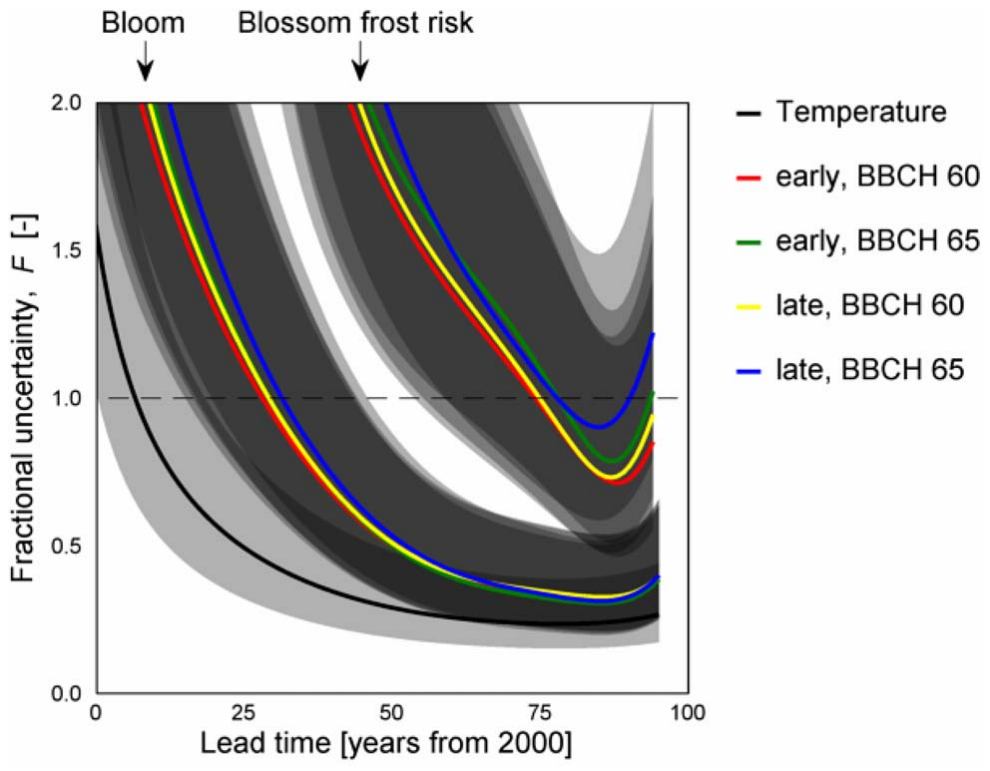
Uncertainty pattern of projected temperature (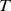), apple bloom (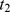) and apple blossom frost risk (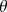). 68.3% percentile (solid lines) and 50-to-90% percentile ranges (gray areas) from 13 climate projections and phenological impact models 2–7 (bloom, blossom frost risk).

The resulting fractional uncertainty 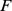 decreased over time. Comparing the sources of uncertainty, the fractional uncertainty of temperature time series decreased faster than of blooming date and blossom frost risk time series. Accordingly, the lowest level of fractional uncertainty at any of the confidence levels investigated was also reached by temperature. While the 90% percentile for temperature and bloom reached 1 in 2019 and 2042–2044 respectively, the uncertainty of blossom frost risk passed 1 only by the 68% percentile (±1 standard deviation) by 2077 (Figure 11). From this point on, the projected change (signal) exceeded the variance of the projection (noise). A minimum of the fractional uncertainty was found for 2078 (temperature), 2083–2084 (bloom) and 2085–2088 (blossom frost risk), after which it was projected to increase. This result was similar for early as well as late ripening varieties and for both BBCH stages.

## Discussion

### Phenological Models

Projections with pure forcing models [20], [21] are subject to changes in dormancy completion [23] and varying warming of the seasons. The application of such a model in the present study produced similar results of increasing risk as in the mentioned literature, but different to the main outcome of the present ensemble study. For this reason, sequential or parallel chilling-forcing models have been recommended [23], as well as models including nearly time-invariant factors as day length [25]. The mean error of all models presented (5.9 d) was in the range of published model performances [15], [20], [21], [23], [25], [42], [43]. This error must be seen in context to the observed flowering duration (BBCH 60 to BBCH 67), which ranged during the calibration period from 6 to 27 d (1 to 99% percentile range). As large errors in simulated flowering dates can erroneously increase the blossom frost risk, the influence of the RMSE on the simulated blossom frost risk was tested (not shown), but no significant influence was found in the range of the calibrated models errors. Having further a negligible bias, the models were rated as suitable for blossom frost risk projections from this point of view. Furthermore, in the present work models were improved by including day length, thus confirming previous findings [25]. Also other models including exponential terms were applied in blossom frost risk estimation [17], [43], relying solely on temperature as input. As they increase the “resistance” for each computation of a day of the year for flowering, exponential models eliminate one deficit of pure temperature sum models which is a calculated flowering date beyond summer in exceptional years, leading to high errors (given that dormancy is completed). In addition, the error of models including a parameter for day length might be lower due to a higher number of parameters. This statistical effect should be separated from the physiological meaning of the parameter. As the role of the length of day in flowering physiology of apple is still under debate [26], these model properties cannot be isolated for the present study, but should be regarded in the future. Finally, while presented combination of sequential or parallel models with an exponential term for day length improves model robustness, these models are also more complex.

### Influence of Climate Change on the Onset of Phenophases

The observed effects of delayed completion of the chilling requirement and earlier flowering due to faster completion of heat requirement are well known[6], [15], [42], [44]–[46]. Thereby the extension of the growing season [47], [48] and the advancement of flowering dates during the past due to climate change have been studied largely for several tree species[44], [49]–[51] including apple flowering phenology [9], [14], [42], allowing the assumption of a general trend. Accordingly “very similar” reactions of apple and cherry blossoming (BBCH 60) as well as winter rye stem elongation (BBCH 31) to early spring conditions were observed [14]. However, the observed mean change of onset of flowering (BBCH 60) of −3.3 d K^−1^ during the short calibration period of phenological models (1991–2012) were lower than those reported from other studies for the entire second half of the 20th century. These published estimates range from −7 to −8 d K^−1^ of year-mean temperatures (values calculated from [9], [42]) for late ripeners up to −5 d K^−1^ of mean temperatures from February to April [14] for early ripeners. Still these discrepancies should result from geographic and orographic differences from the present to the mentioned publications: Analyzing the present model projections for the same periods as in the mentioned literature (1958–2007, 1976–2002, 1969–1998) fairly reproduced these dependencies with −7.5 up to −8.6 d K^−1^ for late, and −6.5 d K^−1^ (February-April temperatures). Consistently, also the projected findings for changes in the onset of apple flowering of −5.4 to −5.6 d K^−1^ (all varieties and stages and years) and −6 d K^−1^ (BBCH 60, February-April temperatures) are in a comparable range. From this can be concluded, that apple flowering phenophases have a clear and comparable reaction to changes in temperature despite differences in region and varieties and that this impact can be tracked by one-dimensional phenological models in combination with climate ensembles.

Furthermore, despite a continuous advancement of flowering dates, an opposing effect of delayed release of dormancy and enhanced spring warming was observed. While warmer winters result in reduced chilling, they can be compensated to a certain extent by warmer springs [52]. For apple bloom this has been reported for the past [42]. However, reduced chilling will eventually slow down the advancement of flowering dates as postulated [42], [52] and as deduced from the relative changes for 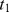 and 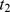 in the present study for the 2nd half of the 21st century. In addition, eventually years with unfulfilled dormancy will occur. Such events have not been observed in Germany during the past century [6], but are discussed for the future [6], [45], [46]. A rough estimate for the probability of years with unfulfilled chilling requirement of up to 15% can be found for the largest producing area in Lower Saxony (Niederelbe) [53]. While this estimate coincides with the here presented range, the mean fraction of years with unfulfilled chilling requirement is lower (3.7%). Following the authors, it must be stated, that these projections are subject to large uncertainties and require further investigation.

### Spring Freeze and Blossom Frost Risk

Last spring freeze follows the warming pattern with changes of increasing speed towards the second half of the 21st century. The projected shifts for the period 1985–2035 (30-year-means) of −2.0 d/decade are in the range of those changes reported for the second half of the 20th century for Central Europe (−2.2 d/decade [12]). Following the future warming pattern in simulations, last spring freeze is likely to change about −3.5 d/decade (2035–2084).

Blossom frost risk possibly decreases in the long term. This result can be obtained roughly by putting together the relative advancement of projected bloom and last spring freezes, as well as in more detail through the present computation with single models. Starting with a blossom frost risk of up to 16%, simulations showed a decline in blossom frost occurrence to about half by the end of the 21st century. Nevertheless, blossom frost is unlikely to disappear and staying at a comparable level as present until the middle of the century. As blossom frost risk strongly depends on the region, period, variety and BBCH stages, publications are hardly comparable. While the present observations and computations for the past are in the range of other studies [9], [19], [20], projected results differ. The often stated hypothesis of an increase in blossom frost risk due to advanced bloom in combination with increased variance in the last spring freeze date [19] does not hold true for the present study, as spring freezes declined comparably faster than flowering dates.

### Projection Uncertainty

Climate impact projection to a near future is often highly uncertain since the internal variability of the system at hand is larger than the expected changes at point of time. As these changes increase with time and relatively to the total variance of the projection, more confidence in the projection signal is gained. Future climate is commonly assessed in ensemble run projections, including RCMs [54] and bias-corrected simulations [35]. Sampling, climate model, radiative and boundary uncertainties have been investigated for climate models, varying for RCMs across field, region and season [54]. While such climate ensembles are also increasingly used to drive impact models [55], the impact models error adds to the signal strength. Uncertainty of climate projections increases with increasing simulation members, as clearly shown by the different patterns of fractional uncertainty of temperature and bloom as well as blossom frost risk. Thereby projection uncertainty of surface temperature depended only on the different climate models, whereas bloom depended on climate and impact models and blossom frost risk additionally depended on the interaction of projected bloom and temperature.

In the present approach times of emergence of 34 years and 57 to 59 years were estimated for temperature and blooming date respectively (compared to the mean 1971–2000), considering one SRES scenario (A1B). This is in the range of the estimated time of emergence for regional surface temperatures of SRES scenarios A2, A1B and B1 from GCMs [39]. While the approach relies heavily on the chosen climate ensemble and impact models, larger variance can be expected with increasing spatial (or temporal) resolution. Therefore the estimated lead time for the minimum of uncertainty of ∼100 years (2078–2088) is consistent with ∼30 to 80 years established for temperature [31]. However, the present works investigated a range of climate and impact models of one scenario, while the cited publications investigated three scenarios for climate models. Hence further projections of future bloom are required in order to remove this lack of comparability. Nonetheless, looking at the cooler scenario B1 and neglecting the similar scenario A2 for central Europe, a larger spread in the day of bloom and hence in the estimated blossom frost risk can be expected, increasing the time of emergence of the climate impact signal. Transferring the estimated time of emergence to other climate impact studies from different research fields by assuming similar variability across models would imply, that a large fraction of these studies operates at the very edge of statistical significance. For example, from a review on 14 publications on future risks through wheat diseases [56], 8 include statements and 2 are solely based on statements for a time horizon ≤2030. From the present findings, the statistical meaning of these studies must be carefully put into context.

Two effects arise: On the one hand, using a location parameter (e.g. mean or median) of a climate ensemble as input for impact models may produce significant future changes while ignoring climate projection uncertainty. On the other hand, using single impact models and/or fixed impact model parameters can give only mean tendencies, similarly ignoring parameter ranges in climate impact. The presented results show these effects, as single impact models with climate ensemble mean as input show consistently significant trends of advancing bloom and, with one exception, of decreasing blossom frost risk. Regarding the total uncertainty of climate and impact models, this may hold true for bloom beyond the estimated projection horizon. However, projected changes in blossom frost risk are low compared to the variability across models. While this is a particularly pronounced problem of extreme events such as blossom frost, it has severe consequences. From the present results, despite a tendency of decreasing blossom frost risk, it must only be concluded that future blossom frost risk is very unlikely to increase.

### Limitations

The present work does not consider the severity and distribution of frosts. Hence it must be taken into account, that other plant reactions than those investigated and resulting from frost distributions may dominate in the future. As actual blossom frost damages were not evaluated, the presented results depict the blossom frost risk tendency. Although blossom frost damage severity increases with decreasing temperature [5], temperatures cannot be translated directly into economic losses, as frost protection (e.g. sprinkler) takes place in practice. Furthermore employed models accounted for day length, but did not use actual surface radiation from climate models. Hence possible effects due to changes in light conditions (e.g. phenological effects) and effects due to severe radiation (radiation frosts) are not represented to full extent. Additionally, the influence of the day length on apple flowering physiology remains uncertain. Despite low availability of consistently bias corrected climate time series of high temporal resolution [29], future approaches should consider this. Finally, future changes in varieties were not taken into account albeit varieties might respond differently to blossom frost [57].

### Conclusions

Regarding the aspects of phenological model structure, simulation uncertainty as well as blossom frost risk, the following conclusions must be drawn from the present findings. Despite a lack of physiological explanation, phenological model performance is improved by including the length of the day. However, projection results from single time series must be put into context to the uncertainty of the modeling chain, considering the significant projection horizon. The latter depends on the investigated variable and was determined for the present simulation of bloom at 2042–2044. Differently, a minimum of uncertainty was estimated for temperature, bloom and blossom frost risk for the range 2078–2088. Finally the resulting regional blossom frost risk cannot be expected to increase in the long term, as compensatory effects of delayed fulfillment of chilling requirement and faster completion of the forcing phase in spring take place.

## Supporting Information

Methods S1 **Basic equations and model formulation.**
(PDF)
