# Supplementary material for: Future Bloom and Blossom Frost Risk for Malus domestica Considering Climate Model and Impact Model Uncertainties
Source: PLoS One. 2013 Oct 8;8(10):e75033. doi: 10.1371/journal.pone.0075033 (PMC3792985; doi:10.1371/journal.pone.0075033)
Supplement: Methods S1 — Basic equations and model formulation. (PDF) [file pone.0075033.s001.pdf]

Future bloom and blossom frost risk for *Malus domestica* considering climate model and impact model uncertainties

Holger Hoffmann[1]*, Thomas Rath,
Biosystems Engineering, Institute for Biological Production Systems, Leibniz Universität Hannover, Hannover, Germany
[1] Present address: Institute of Crop Science and Resource Conservation, University of Bonn, Bonn, Germany
* E-mail: Corresponding hhoffmann@uni-bonn.de

Phenological models supplementary S1

Projection of temperature

Temperature time series are presented as anomaly from the 1971-2000 mean as indicated by ΔT. The anomaly of single years as well as of 10 year moving average time series is shown. By way of example, the latter was calculated as:

$$\Delta T_{y1,y2,s} = \frac{1}{10}\sum_{i=-4}^{5}\frac{1}{n}\sum_{d=1}^{n}T_{y2+i,d,s} - \frac{1}{30}\sum_{i=-14}^{15}\frac{1}{n}\sum_{d=1}^{n}T_{y1+i,d,s} \text{ with} \quad (1)$$

$\Delta T_{y1,y2,s}$: projected change in year-mean air temperature from year $y1$ to year $y2$ of every grid point s in Lower Saxony, [-]
$y1, y2$: year of calculation (past, future)
s : grid point
i : index
d : day
n : number of days of the year (365 or 366)

Projection of bloom

The change in blooming date Δt_2 was calculated as the difference in the 30-year-mean for each grid point:

$$\Delta t_{2y1,y2,s} = \frac{1}{30}\cdot\sum_{i=-14}^{15}t_{2y2+i,s} - \frac{1}{30}\cdot\sum_{i=-14}^{15}t_{2y1+i,s} \text{ with} \quad (2)$$

$\Delta t_{2y1,y2,s}$: projected change in blooming date t_2 from year $y1$ to year $y2$ of every grid point s in Lower Saxony, [-]
$y1, y2$: year of calculation (past, future)
s : grid point
i : index

Years with unfulfilled chilling were recorded by counting years without bloom or bloom projected for DOY>200 as fraction of occurrences in a 30-year-mean:

$$\chi_y = \frac{1}{30} \cdot \sum_{i=-14}^{15} \mu_i \text{ with} \quad (3)$$

$$\mu_i = \begin{cases} 1 & \text{if} \quad t_{2,y+i} > 200 \\ 0 & \text{else} \end{cases}$$

χ : Fraction of years with unfulfilled chilling requirement, [-]

$t_{2,y}$: onset of phenophase in year y, [DOY]

y : year of calculation, e.g. 1980

i : index

Calculation of probability mass functions

The values of probability mass functions were estimated non-parametrically by applying a Gaussian kernel:

$$pdf(x) = \sum_{s=1}^{n} \frac{1}{nh\sqrt{2\pi}} e^{-\frac{(x-\Delta\theta_{y1,y2,s})^2}{2h^2}} \text{ with} \quad (4)$$

$$h = 0.03$$

$pdf(x)$: probability density function value over all grid points, [-]

$\Delta\theta_{y1,y2,s}$: projected change in blossom frost risk

x : any possible value of $\Delta\theta_{y1,y2,s}$, [-]

h : bandwidth of kernel smoothing window, [-]

s : grid point

n : number of grid points

$$pmf(x) = \frac{pdf(x)}{\sum_{j=1}^{z} pdf(j)}, \text{ with} \quad (5)$$

$pmf(x)$: probability mass function value over all grid points, [-]

z : number of possible values of $\Delta\theta_{y1,y2,s}$, [-]

j : index

Model description

Apple bloom was simulated using phenological models. In principle, models assume that the time of bloom is related to so-called temperature sums of chilling (Sc) and forcing (Sf), accumulated during winter (chilling phase) and spring (forcing phase) by the corresponding rates of chilling (Rc) and forcing (Rf). See tab. 1 for denominations.

$$Sc(t) = \sum_{i=t_0}^{t} Rc(T_i) \quad (6)$$

$$Sf(t) = \sum_{i=t_1}^{t_2} Rf(T_i) \quad (7)$$

Further it is assumed, that Sf is related to Sc as follows:

$$\text{Sequential models: } Sf(t_2) = a \cdot e^{bSc(t_1)} \quad (8)$$

$$\text{Parallel models: } Sf(t_2) = a \cdot e^{bSc(t_2)} \quad (9)$$

A basic thermal-time model (model 1) was applied as described, with the rate of forcing Rf:

Model 1

$$Rf(T_i) = \begin{cases} 0 & \text{if} \quad T_i \leq Tbf \\ T_i - Tbf & \text{else} \end{cases} \quad (10)$$

Sequential (model 2) and parallel (model 3) chilling-forcing models were applied as described in the following:

Models 2,3

$$Rc(T_i) = \begin{cases} 0 & \text{if} \quad T_i \leq 0 \text{ or } T_i \geq 10 \\ \frac{T_i}{Tbc} & \text{if} \quad 0 < T_i \leq Tbc \\ \frac{T_i - 10}{Tbc - 10} & \text{if} \quad Tbc < T_i < 10 \end{cases} \quad (11)$$

$$Rf(T_i) = \begin{cases} 0 & \text{if} \quad T_i \leq Tbf \\ \frac{28.4}{1+e^{(-0.185(T_i - Tbf - 18.4))}} & \text{else} \end{cases} \quad (12)$$

The Modified Utah model was applied for mean daily temperature values (model 4). Following a different approach, this model is a sequential model with Rc as in eq. 11 and with Rf being:

Model 4

$$Rf(T_i) = \begin{cases} 0 & \text{if} \quad T_i \leq Tbf \\ (T_i - Tbf) \cdot \left[1 + \left(\frac{Sf(T_{i-1})}{Sf(t_2)}\right)^2\right] & \text{else} \end{cases} \quad (13)$$

Due to findings for better performance when relating bloom additionally to radiation, models taking into account the length of the day were further included (models 5-7). Model 5 was applied in the version described, and being an extension of model 1 the rate of forcing is calculated as follows:

Model 5

$$Rf(T_i) = \begin{cases} 0 & \text{if} \quad T_i \leq Tbf \\ (T_i - Tbf) \cdot \left(\frac{D}{10}\right)^c & \text{else} \end{cases} \quad (14)$$

Models 6-7 are new variations of the sequential and parallel chilling-forcing models. These varied models also assume, that bloom is influenced by radiation only during the forcing phase. For both Rc was calculated as in eq. 11 and Rf was calculated as follows:

Model 6,7

$$Rf(T_i) = \begin{cases} 0 & \text{if} \quad T_i \leq Tbf \\ \frac{28.4}{1+e^{(-0.185(T_i - Tbf - 18.4))}} \cdot \left(\frac{D}{10}\right)^c & \text{else} \end{cases} \quad (15)$$

Table 1. Denomination of variables and parameters

Notation	Description	Unit
T	Air temperature	°C
Tbc, Tbf	Base temperature for chilling, forcing	°C
t	Time	hour [h], day [d] or year [a]
t_0	Start of the chilling period (dormancy)	day of the year (DOY)
t_1	Chilling requirement completed, start of forcing	day of the year (DOY)
t_2	Forcing completed (BBCH 60, BBCH 65)	day of the year (DOY)
Sc, Sf	State of chilling, state of forcing	—
Rc, Rf	Rate of chilling, rate of forcing	—
D	Daylength	h
a, b, c	Calibration parameters	—
i, s, z	Index variables	—
θ	Blossom frost risk	—
β	Temperature threshold for blossom frost	°C

Model parameters

Table 2. Model parameters (early ripeners, BBCH 65, area mean)

Model	Tbc [°C]	Sc [-]	Tbf [°C]	a [-]	b [-]	c [-]	t_1 [DOY]	t_2 [DOY]
1	—	—	5.8	—	—	—	—	122.6
2	3.0	36.9	5.0	220.9	-0.0248	—	12.1	120.4
3	2.5	37.8	3.1	201.3	-0.0029	—	17.4	121.9
4	4.2	37.7	7.4	—	—	—	17.4	121.9
5	—	—	0.7	—	—	1.3	[a]30.1	122.9
6	4.8	33.4	5.2	232.1	-0.0063	4.4	3.0	120.4
7	5.1	35.7	5.7	215.9	-0.0033	5.7	8.0	119.2

[a]This model does not calculate the fulfillment of dormancy, but optimizes t_1 as starting date for heat summation.
